# Evaluating ethics oversight during assessment of research integrity

**DOI:** 10.1186/s41073-019-0082-6

**Published:** 2019-11-06

**Authors:** Andrew Grey, Mark Bolland, Alison Avenell

**Affiliations:** 1 Department of Medicine, University of Auckland, Private Bag, Auckland, 92019 New Zealand; 2 University of Aberdeen, Aberdeen, UK

**Keywords:** Ethics, Integrity, Institutional investigation

## Abstract

We provide additional information relevant to our previous publication on the quality of reports of investigations of research integrity by academic institutions. Despite concerns being raised about ethical oversight of research published by a group of researchers, each of the four institutional investigations failed to determine and/or report whether ethics committee approval was obtained for the majority of publications assessed.

Recently, we reported that four institutional investigations of the integrity of publications between 1996 and 2013 by a single research group were of low quality [1]. Three institutions were in Japan (institutions 1, 2, and 3) and one in the USA (institution 4). Among the many concerns raised with the institutions was uncertain ethics oversight. Surveys in Japan reported that by 1992 all 80 medical schools had established ethics committees and by 1995 93% of medical schools’ ethics committees were reviewing clinical research with patients as participants [2, 3].

In the reports of their investigations, none of the institutions addressed the concerns about ethics oversight. In correspondence with one of us (AG), institution 1 stated that 4 of 38 papers it assessed (all retracted) had been approved by its ethics committee but did not report whether the other papers had received ethics committee approval. Neither institution 2 nor institution 4 mentioned evaluation of ethics oversight for any papers. Institution 3 reported the findings of its investigation after submission of our paper. It assessed 40 publications, but did not mention ethics oversight in its report or correspondence with us.

Overlapping the institutional investigations, two journals which had published research by the group in question between 1996 and 2001 that claimed ethics committee approval established that there was no ethics committee in place at that time at either institution 3 or one of the hospitals at which research was conducted by staff affiliated to institution 3 [4–6]. Thus, the investigation by institution 3 failed to determine that research conducted at its own facilities was unethical.

Unethical conduct of research is a serious breach of research integrity and grounds for retraction [7]. Ethics oversight should routinely be addressed during assessment of research integrity by journals and institutions. Institutional investigations should evaluate and report the ethics committee (name, reference number and date of review) responsible for each piece of work assessed. Documentary evidence of ethics oversight could be incorporated into the journal manuscript submission process. In the specific case described herein, we suggest that journals, publishers and institutions who are considering concerns about the integrity of the work seek evidence that it was conducted with adequate oversight of ethics.

## Data Availability

No original data are included in the manuscript.
